# Cell Cycle-Dependent Localization of Voltage-Dependent Calcium Channels and the Mitotic Apparatus in a Neuroendocrine Cell Line(AtT-20)

**DOI:** 10.1155/2009/487959

**Published:** 2010-01-06

**Authors:** Karen J. Loechner, Wendy C. Salmon, Jie Fu, Shipra Patel, James T. McLaughlin

**Affiliations:** ^1^Division of Pediatric Endocrinology, Department of Pediatrics, University of North Carolina Chapel Hill, Chapel Hill, NC 27599, USA; ^2^Michael Hooker Microscopy Facility, University of North Carolina Chapel Hill, Chapel Hill, NC 27599, USA; ^3^Department of Pharmacology, University of North Carolina Chapel Hill, Chapel Hill, NC 27599, USA

## Abstract

Changes in intracellular calcium are necessary for the successful progression of mitosis in many cells. Both elevation and reduction in intracellular calcium can disrupt mitosis by mechanisms that remain ill defined. In this study we explore the role of transmembrane voltage-gated calcium channels (CaV channels) as regulators of mitosis in the mouse corticotroph cell line (AtT-20). We report that the nifedipine-sensitive isoform CaV1.2 is localized to the “poleward side” of kinetechores during metaphase and at the midbody during cytokinesis. A second nifedipine-sensitive isoform, CaV1.3, is present at the mid-spindle zone in telophase, but is also seen at the midbody. Nifedipine reduces the rate of cell proliferation, and, utilizing time-lapse microscopy, we show that this is due to a block at the prometaphase stage of the cell cycle. Using Fluo-4 we detect calcium fluxes at sites corresponding to the mid-spindle zone and the midbody region. Another calcium dye, Fura PE3/AM, causes an inhibition of mitosis prior to anaphase that we attribute to a chelation of intracellular calcium. Our results demonstrate a novel, isoform-specific localization of CaV1 channels during cell division and suggest a possible role for these channels in the calcium-dependent events underlying mitotic progression in pituitary corticotrophs.

## 1. Introduction

Voltage-dependent calcium channels (CaV channels) are multisubunit transmembrane proteins that are mediators of entry of extracellular calcium ions into cells of nerve, muscle, and endocrine tissues [1]. Genomic studies have identified 3 families for the ten genes that encode the alpha1 subunits designated CaV1, CaV2, and CaV3 [2]. The diversity of CaV channel genes allows for a large number of channel isoforms, and these different isoforms are often expressed in the same cell. By mediating changes in intracellular free calcium, CaV channels act as key mediators of signaling events such as cell depolarization, neurotransmitter and neuropeptide secretion, and regulation of gene expression [3, 4]. An important objective in calcium channel biology, therefore, is to understand the specific role(s) for each channel isoform, and their integration in different cellular events. 

 One well-established role of calcium channels is the coupling of membrane depolarization to release of neurotransmitters [5, 6]. Both CaV2.1 and CaV2.2 have been shown to interact directly with, and be modulated by, proteins that comprise the neurotransmitter release apparatus (the SNARE complex). Colocalization of channels and the release machinery facilitates coupling between the active calcium channels and the calcium dependent fusion of transmitter-containing vesicles with plasma membrane. 

In neuroendocrine cells, a similar coupling between CaV1 channels and release machinery is thought to underlie secretion of peptides such as insulin, growth hormone, or ACTH [7, 8]. The pituitary corticotroph cell line, AtT-20, is a well-established model system for studies of ACTH secretion. These cells express multiple isoforms of CaV, yet only the CaV1 channels are coupled to CRH- or depolarization-stimulated secretion of ACTH [9, 10]. In a recent study we examined the cellular distribution of CaV1 channels and SNARE proteins in AtT-20s cells and found colocalization of CaV1.2, but not CaV1.3, with components of the synaptic machinery and releasable peptide [11]. In the course of this study we observed CaV1 channels localized near components of the mitotic apparatus in dividing cells. These observations suggested that the AtT-20 cell could provide a useful model to examine the possible role for CaV1 channels in another cellular function, mitosis. 

 A role for calcium signaling in mitosis has been inferred for decades, yet the mechanism underlying calcium elevation during cell division has, to date, not been elucidated. Studies have established a role for calcium and/or demonstrated alterations in calcium gradients during mitosis [12–19]. Calcium is involved in regulating mitotic checkpoints; the critical point at which progression through mitotic stages is closely regulated has also been shown [20–24]. Furthermore, the role of calcium-dependent kinases in mitosis has also been examined (reviewed in [25]). 

 The CaV channels observed in dividing AtT-20 cells represent a possible contributor to intracellular calcium fluxes during mitosis. Antagonists to the CaV1 subtype (dihydropyridines (DHPs)) have been reported to block mitosis in a number of systems [26–29], as so it is possible that these channels play a role in the mitotic process. While the limited access to the mitotic apparatus could present a barrier to some drugs, DHPs are highly lipophilic [30] and therefore could reach internal membrane sites. In this paper, we show that CaV1 channels are localized in close proximity to mitotic structures. We then use several experimental approaches to assess whether these channels are active participants in cell division in AtT-20 cells.

## 2. Methods

### 2.1. Cell Culture

AtT-20/D16v cells are from American Type Culture Collection (ATCC, Manassas, VA). All cell culture reagents are obtained from Invitrogen (Carlsbad, CA). AtT-20 cells are cultured in Dulbecco's Modified Eagle medium (DMEM) containing 4.5 gm glucose/L and 10 percent fetal bovine serum. Cells are maintained at 37°C in a 5% CO2 humidified incubator and passaged as described previously [9]. For microscopy, cells are plated on chambered cover slips (Labtek/Nunc, Rochester NY) and grown 2 to 4 days prior to fixation. For proliferation assays, cells are plated in 8-well plates (for cell counting) and 96-well plates (for MTS assay) to be used 2–4 days after plating (~60%–90% confluence). For calcium and time-lapse imaging, cells are plated 2–4 days prior to experiments on coated glass bottom microwell dishes (Mattek Corp, MA). Cells are then transferred to steroid-free media (Charcoal/Dextran treated FBS; Hyclone, Logan UT) 12–18 hours prior to fixation.

### 2.2. Immunocytochemistry

Cells are rinsed briefly in Phosphate-Buffered Saline (PBS), and then fixed by incubation 20 minutes in PBS containing 4% paraformaldehyde. Following three washes with PBS, cells are incubated 5 minutes in 100% methanol at 20°C. They are washed again in PBS, and then permeabilized in PBS containing 20 mM NaN3, 0.2% Tween-20, and 0.5% NP-40. All subsequent washes employ Antibody Wash Buffer (WB; PBS plus 0.2% Tween-20 and 0.05 mg/mL Bovine serum albumin (BSA, IgG free; Sigma, St. Louis MO). Fixed cells are incubated 24–48 hours at 4°C with one or more primary antibodies diluted into WB (antibody sources listed below). Following 5 washes with WB, cells are incubated with the appropriate combination of fluorescent secondary antibodies (Alexa-fluor conjugates, Invitrogen, Carlsbad CA) for 1 hour at 22°C. This is followed by 3 washes, and a final rinse with PBS. Stained cells are stored at 4°C in Slo-fade (Invitrogen); or in Slo-fade containing the nuclear counterstain DAPI (4′-6-Diamidino-2phenylindole; Invitrogen). Primary antibodies are obtained from the following commercial sources: Antibodies to CaV1 channel alpha1 subunits are from Alomone Labs (Jerusalem, Israel); Anticentromere (CREST) antibody was from Antibodies Incorporated (Davis, CA); anti-*α* tubulin, clone DM1A from Sigma (St. Louis, MO). The antibodies to CaV1 channel alpha1 subunits are raised in rabbits against peptide sequences specific to the CaV1.2 (peptide sequence: TTKIN MDDLQ PSEN EDKS) and CaV1.3 (peptide sequence: DNKVT IDDYQ EEAE DKD) isoforms. A basic local alignment search (Blast; National Center for Biotechnology Information, http://www.ncbi.nlm.nih.gov/) of all non-redundant protein sequence databases shows no known protein other than these with greater than 50% sequence similarity to either of these sequences; similar searches of the mouse protein database identify the peptide sequences only in these two channel polypeptides. In tests of specificity, we detect no specific CaV1 staining when the primary antibody is omitted from the protocol, or when the antibody is preadsorbed to a ~10-fold molar excess of peptide antigen (not shown).

### 2.3. MTS Assay

Cells are plated at low density in 96-well plates (5 × 10/well), allowed to grow for 24–48 hours, and then incubated 72 hours in media to which nifedipine is added at the concentrations indicated. All wells contain the same concentration of vehicle, 0.05% DMSO. Cell density is measured using a modified methyl tetrazolium protocol (MTS; Promega Madison WI) according to manufacturer's instructions. Data is normalized to absorbance of cells incubated with vehicle alone and plotted as a function of nifedipine concentration. All graphs are plotted using Origin v8 (OriginLab, Northampton MA).

### 2.4. Confocal Microscopy

**Figure 1:**
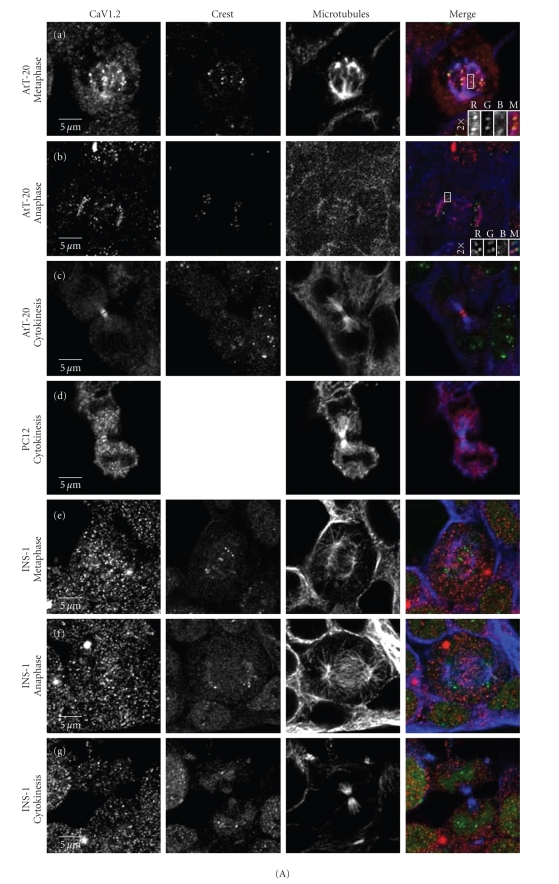

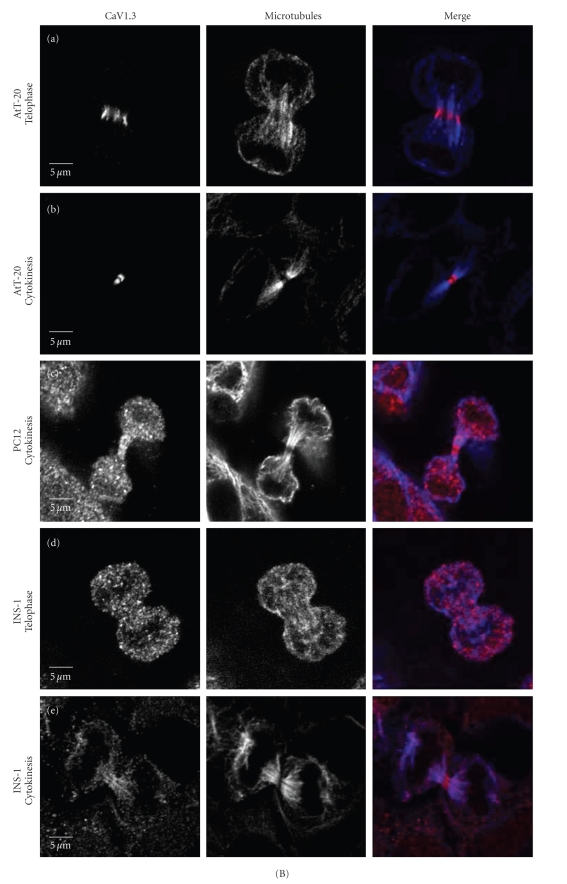

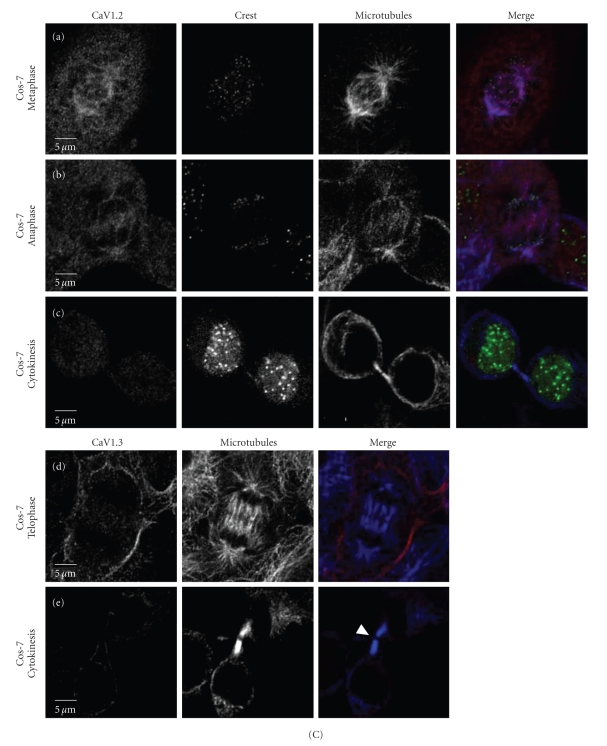
*Calcium channel *CaV1.2* and *CaV1.3* localization in mitotic cells*. 1(A) compares CaV1.2 staining in AtT-20 cells (a)–(c), PC-12 cells (d), and INS-1 cells (e)–(g). CaV1.2 is located with kinetechores at the “poleward side” during metaphase and anaphase ((a), (b): inset) in the AtT-20 cells. Midbody CaV1.2 staining is observed in both AtT-20 cells and PC-12 cells (c), (d) but not in INS-1 cells (g). 1(B) is a similar comparison for CaV1.3 and shows staining at the mid-spindle zone only in AtT-20s during telophase (a). In contrast, all three neuroendocrine cell types display CaV1.3 staining at the midbody during cytokinesis ((B): (b), (c), and (e)). 1(C) shows control staining of Cos 7 cells; in particular, note that there is no nonspecific staining at the midbody of these cells (1(C) (c), (e)). CaV1.2 and CaV1.3 are stained with antibodies specific to alpha subunit sequences for CaV1.2 and CaV1.3 (see Methods, Alamone Labs) and detected with secondary goat anti-rabbit antibody Alexa 555 (red); Microtubules are stained with DM1A and goat anti-mouse Alexa 647 (blue); kinetechores are stained with anti-CREST antibody and Alexa 488 (green). Bleedthrough between microtubule and CREST staining channels was corrected for as described in methods.

We image stained cells at multiple wavelengths using a Zeiss 510 Meta laser scanning confocal with inverted microscope stand (Carl Zeiss, Jena, Germany) using a 63X 1.4NA oil immersion objective. Mitotic stages are detected by eye using DAPI to visualize chromosome morphology; due to the limitation of the confocal system to 3 color channels, imaging of chromosomes was not possible in addition to the proteins described. All channels are imaged sequentially, and images are stored as chunky RGB files. Figures are assembled using Adobe Photoshop (ver. 7.0). Images with four labels (Figure 1(A)) display some bleedthrough of the microtubule staining into the CREST staining. We corrected for this using MetaMorph v4.5 software (Molecular Devices, Sunnyvale CA) by calculating the relative intensity of microtubules in both the CREST and microtubule images, multiplying the microtubule image by the value and subtracting the resultant image from the CREST image.

### 2.5. Visual Mitotic Stage Assay

Cells are plated at low density (5 × 10^3^/well) in chambered cover slips (Labtek/Nunc, Rochester NY), allowed to attach for 24–48 hours, and then incubated either 24 or 72 hours in media containing Nifedipine (6 *μ*M). Multiple fields (minimum of 5 per condition) of AtT-20 cells at similar cell density are randomly selected and imaged with a Hamamatsu ORCA-ER camera mounted on a Leica DMIRB inverted microscope equipped with a mercury lamp and DAPI filter cube and 40X 1.25NA objective. The number of cells in each stage is determined using “Manually Count Objects” application in MetaMorph. We group cells identified in anaphase, telophase, or cytokinesis into a single class (A/T/C) in our analysis due to the low mitotic index and the difficulty in differentiating among these phases using chromosome morphology alone.

### 2.6. Time-Lapse Microscopy

Time series of DIC images are acquired as described above using a 30-second time interval. Cells are maintained in covered dishes at 37°C over the course of the imaging session to minimize changes in osmolarity. We define a significant mitotic “block” as any stage in which time elapsed is equal to or greater than two standard deviations of the mean time required for that stage by control cells. For Fura PE3/AM AtT-20 cells are imaged by DIC on a Nikon TE2000U inverted microscope with a 100X 0.5–1.3NA S Fluor objective with the iris collar set to 1.3NA using a DVC 1312 monochrome CCD camera (DVC Co.) controlled with Simple PCI software (v5.3, Hamamtsu, Inc.). Cells are kept at 37°C using an air current incubator (AirTherm). To ascertain that the effects on cell cycle progression are not a function of the imaging setup or cell culture conditions, a prophase cell is imaged for the entire mitotic cycle (~1.5 hours) without the addition of Fura PE3/AM (4 *μ*M) on each experimental day. We then exclude all data collected on days when the control cells do not complete the entire mitotic cycle within the mean time ±1 S.D. Experimental dishes are incubated with Fura PE3/AM (4 *μ*M) at 37°C for 20 minutes prior to DIC imaging of mitosis versus vehicle controls (DMSO).

### 2.7. Calcium Imaging

Fluo-4 fluorescence is imaged in live cells on a Nikon TE2000U inverted microscope a DVC 1412 Intensicam II. The DIC and fluorescence images are acquired simultaneously using a long pass 610 nm filter for the DIC illumination, a dual camera adapter connected to the camera port, and a 565 nm dichromatic mirror in the dual-camera adapter to direct the fluorescence emission light to the DVC 1412 Intensicam II and the DIC image to a DVC 1312 camera. A Sutter DG-4 xenon arc lamp source and fast filter changer are used for illumination of fluorescence and selection of excitation wavelengths. An FITC filter set is mounted in the microscope stand with white light delivered from the DG-4. Fluorescence illumination and all image acquisitions are controlled using the SimplePCI image acquisition software running on two PC computers, one for each camera. To minimize phototoxicity to the cells from excessive fluorescence excitation, every one minute a set of 10 images is acquired at a 1 second interval. The rapidity of the acquisition pattern is due to the short duration of anaphase (minutes). Signal intensity of multiple areas in the time-lapse image series is determined using the intensity measurement functions in the SimplePCI software.

## 3. Results

### 3.1. CaV1.2 and CaV1.3 Localize to the Mitotic Apparatus

Figures 1(A) and 1(B) show the pattern of staining observed for CaV1.2 and CaV1.3, respectively, for AtT-20 cells captured and fixed at different stages in mitosis. To visualize the kinetechores in metaphase, cells are stained with a kinetechore antibody (CREST antibody) in addition to antibodies to CaV1.2 and CaV1.3. Costaining demonstrates that CaV1.2 localizes to sites adjacent to the “poleward” of the kinetechores in both metaphase and anaphase (Figures 1(A), (a), (b), inserts). Of note, this distribution of CaV1.2 with kinetechores during metaphase is seen throughout the volume of the cell as imaged using z-stacking across the entirety of the spindle apparatus (not shown) and supports that the localization is within the AtT-20 cell and not superimposed imaging from surface proteins. 

 To visualize the microtubules of the mitotic spindle, cells are also costained with the *α*-tubulin antibody DM1A. In telophase, we observed CaV1.3 (Figure 1(B), panel (a)) at the mid-spindle zone of the mitotic apparatus; we do not observe a similar pattern with CaV1.2 (Figure 1(A), panel (a)). However, as the microtubules condense further to form the midbody during cytokinesis, both CaV1.2 and CaV1.3 are then visualized at the midbody (Figure 1(A) panel (c), and Figure 1(B) panel (b)). These staining patterns suggest that the cells direct an active, isoform-specific redistribution of CaV1 channels over the course of the cell division cycle. 

 To test if the mitotic channel distribution occurs in other cell types, we examined the distribution of CaV1 channels in two additional cell lines of neuroendocrine origin: PC12 pheochromocytoma cells and INS-1 beta pancreatic cells. Both of these cell types are known to express CaV1 channels [31, 32]. Figure 1 shows that, as in AtT-20 cells, both CaV1.2 (Figure 1(A) panel (d)) and CaV1.3 (Figure 1(B) panel (c)) are localized to the midbody in PC12s during cytokinesis. In contrast, we did not see a similar pattern of CaV1 staining in INS-1 cells undergoing mitosis; the CaV1.3 channels are visible at midbody whereas the CaV1.2 channels are not (Figure 1(A), panel (g); Figure 1(B), panel (e)). Specific staining for both isoforms was readily visible in interphase cells as reported previously [31]. These differences in channel isoform staining support the idea that the patterns we observe are due to real differences among neuroendocrine cell types, and not simply a nonspecific antibody interaction with mitotic structures. 

 As another test of specificity we performed the identical imaging protocol using the epithelial-derived Cos 7 cells that lack CaV1 channels [33, 34]. Figure 1(C) shows that in these cells we detect only background staining suggestive of nonspecific binding of the antibodies; no pattern specific to the mitotic apparatus is seen.

### 3.2. Incubation with CaV1 Antagonist, Nifedipine, Attenuates Cell Proliferation

**Figure 2:**
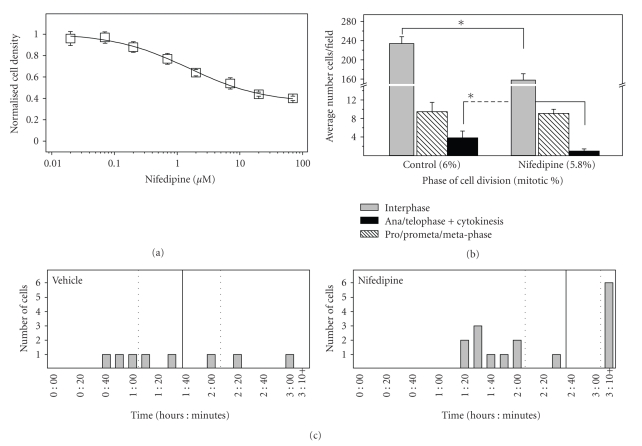
*Nifedipine attenuates AtT-20 Cell density*. (a) *Cell viability*. Incubation with the DHP CaV antagonist, nifedipine, results in a dose-dependent decrease in AtT-20 cell number using the colorimetric MTS assay for cell viability. The half maximal dose for this effect is 1.5 ± 0.3 *μ*M nifedipine. Graph shows normalized data from 3 experiments. (b) *Cell proliferation and mitotic stages.* Graph shows mean number of cells ±SE for at least 5 fields (≥ 800 total cells counted for Day 3 for each condition). There is a significant decrease in the total number of cells counted between vehicle control and nifedipine-treated cells (*P < .05). There is also a parallel decrease in later phases of mitosis (A/T/C) (*P < .05). Nifedipine = 6 *μ*M; Vehicle = DMSO from which nifedipine is diluted from 10 mM stock solution. (c) *Time for transition through mitosis.* Measurements for individual AtT-20 cells using time-lapse microscopy. The time from prophase to cytokinesis is depicted for both control (Vehicle) and Nifedipine-treated cells. The mean time for completion of mitosis is indicated by the bold line with the dotted lines representing 2 S.D. from the mean. Nifedipine treated cells show a shift in mean time to complete mitosis as well as an increased number of cells that do not complete mitosis (block) within the 2 S.D. time limit compared to control cells.

To test for a potential role for CaV1 channels in mitosis, we examined the effect of the dihydropyridine (DHP) CaV1 antagonist, nifedipine, on cell growth using a standard assay of cell viability (MTS; see Methods). Cells are cultured in the presence of varying concentrations of nifedipine for ~72–96 hours. The effect of nifedipine on cell division was assessed by measuring cell density following this incubation. We used this prolonged incubation to amplify any potential effect of nifedipine on cell division. Figure 2(a) shows a dose-dependent attenuation of cell growth by nifedipine. The maximal effect approaches a 60% decrease in cell density after three days of incubation, corresponding to an approximately 2.5-fold decrease in growth rate. The IC50 for this effect is approximately 1.5 *μ*M, a value that is substantially higher than the IC50 measured for DHP-induced blockade of ACTH release [9]. While the role of the CaV1 isoforms 1.2 and 1.3 in secretion has not been unequivocally established, recent work in INS-1 cells demonstrates that only the CaV1.2 isoform is coupled to secretion [31]. In addition, there is evidence that the CaV1.3 channel has a lower affinity for DHPs than the CaV1.2 isoform [35], and thus may represent the target responsible for effects of nifedipine on cell proliferation we observe.

### 3.3. Incubation with Nifedipine Results in an Apparent Shift in Mitotic Stage

We next examined the effect of nifedipine on cell proliferation using a direct cell counting assay. Treatment of fixed AtT-20 cells with the nuclear stain (DAPI) allows us to identify cells at various mitotic stages according to chromosome morphology. To maximize our ability to detect a DHP-sensitive effect on mitotic cells given the small percentage of cells undergoing cell division at any one time (4%–6%), we counted an average of 800 cells/condition using at least 5 different fields imaged (shown as the average no. cells/field). First, we observed a significant decrease in cells per field in the presence of nifedipine (Figure 2(b), Control versus Nifedipine, *P* < .05). Second, although the total percentage of cells undergoing mitosis is essentially unchanged in the presence of nifedipine (4%–6%), within the mitotic population we observe a significant decrease in the percent of A/T/C cells (Anaphase/Telophase/Cytokinesis; Figure 2(b), *P* < .05). This shift, along with the observed cumulative decrease in cell number over time in nifedipine, is indicative of a block in mitosis that occurs prior to the cell entering anaphase. The cumulative nature of the effect suggests that as additional cells enter the mitotic cycle, more stall and are unable to proceed to complete cell division. We also do not detect any obvious change in spindle morphology in response to incubation with nifedipine. Finally, we believe that this decrease is likely due to an effect on cell division and not on apoptosis in that we find no evidence for either DNA laddering or other markers of apoptosis in nifedipine-treated AtT20 cells (not shown). Calcium channel block is generally associated with a protective effect in models of apoptotic cell death [36].

### 3.4. Nifedipine Induces a Phase-Dependent Block in Mitosis

As another approach to examining the effects on nifedipine on mitosis, we next used a direct time-lapse microscopy assessment of individual cell division. AtT-20 cells are imaged in the presence of 6 *μ*M nifedipine (or vehicle control, DMSO) at different stages (Interphase through Cytokinesis) to determine the average time spent in each mitotic stage under these two conditions. Figure 2(c) shows the effect of nifedipine on the progression of cells in prophase/prometaphase/metaphase to anaphase. Nifedipinetreated cells display a block in mitotic progression (52%; *n* = 23 cells) versus control cells (15%; *n* = 26 cells; *P* ≤ .05). The magnitude of this DHP-induced block can be expressed as a “mean time to complete mitosis” (vertical line ±2 standard deviations, dotted lines). The mean time from prophase to cytokinesis is shifted in nifedipine treated cells (bottom panel) although there is an overlap between the two groups. In other words, both control and nifedipine treated cells can “stall” in prometaphase/metaphase, but this phenomenon occurs more frequently in the presence of nifedipine. In cells observed over a prolonged time course we observe cells undergoing chromosome decondensation and reversion to interphase. Finally, although cell death could be observed, it is seen in both control and nifedipine-treated cells, particularly those imaged over a longer time course (e.g., hours).

### 3.5. Calcium Chelation Disrupts Mitosis

**Figure 3:**
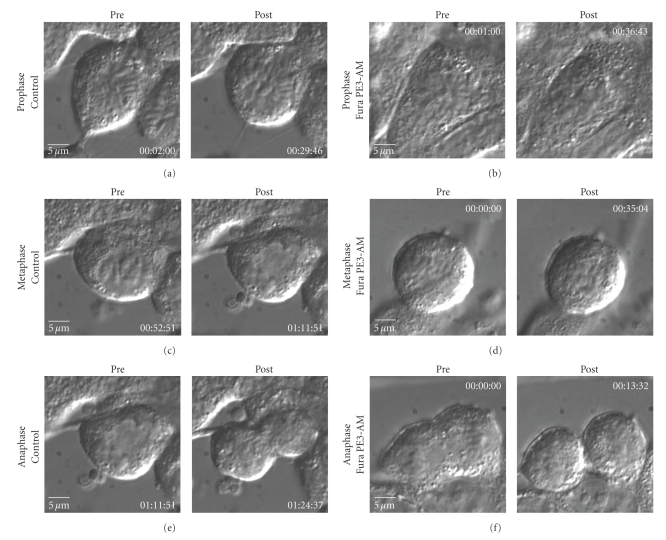
*Calcium chelation with fura PE3/AM interrupts specific stages of mitosis*. Addition of Fura PE3/AM (4 *μ*M) results in a cessation and/or regression of mitotic stages prophase (b) and metaphase (d). However, progression from anaphase to cytokinesis is unaffected (f). (a), (c) and (e) are controls for comparison; images are captured at 30-second intervals as described in Methods; times for these images are as indicated in lower right of each panel. In the control cell (a) prometaphase proceeds to metaphase within 51 minutes and continues to cytokinesis within 1 minute 18 seconds. In contrast, in the Fura PE3/AM-treated prometaphase cell (b), chromosomes decondense and no mitotic progression is seen in >60 minutes. Similarly, in the presence of Fura PE3/AM, metaphase cells show chromosomes aligned but do not progress in >30 minutes (d). In the presence of Fura PE3/AM, anaphase progresses normally to cytokinesis (f).

We next examined the effects of several calcium-sensitive dyes, both for their potential effects on mitosis (due to calcium chelation) and as a tool to detect calcium transients in mitotic AtT-20 cells. As reported in other systems [13, 14], we find that treatment of AtT-20 cells with the calcium indicator Fura PE3/AM (4 *μ*M) disrupts mitosis at all stages prior to anaphase (Figures 3(b), 3(c)). In contrast, if Fura PE3/AM is added to a cell already in anaphase/telophase, mitosis proceeds without obvious delay (Figure 3(d)). To ascertain if diffuse cell injury was occurring in response to chelation of intracellular calcium by the presence of Fura PE3/AM, we imaged interphase cells (or “nondividing”) to observe if spontaneous action potentials that can be seen in AtT-20 cells could be observed by measuring the concomitant calcium transients. As seen in Figures 3(b) and 3(c), relative fluorescence corresponding to calcium spikes detected using Fura PE3/AM could be measured in a nearby interphase cell (arrow), showing that the cells could still exhibit electrical activity after Fura PE3/AM loading; this is displayed graphically in Figure 3(c). The effect of Fura PE3/AM is, therefore, consistent with published reports demonstrating that perturbation of intracellular calcium can lead to disruption of mitosis. This “chelation effect” is distinct from the changes in intracellular calcium levels that may occur due to CaV1, particularly in nondividing cells. However, it is provocative that both calcium chelation and DHP-induced blockade of CaV channels result in a prometaphase block in mitosis, suggesting that the intracellular calcium requirement for mitosis may be uniquely relevant to this stage in mitosis.

### 3.6. Calcium Transients Colocalize with Distribution of CaV1.2 and CaV1.3

**Figure 4:**
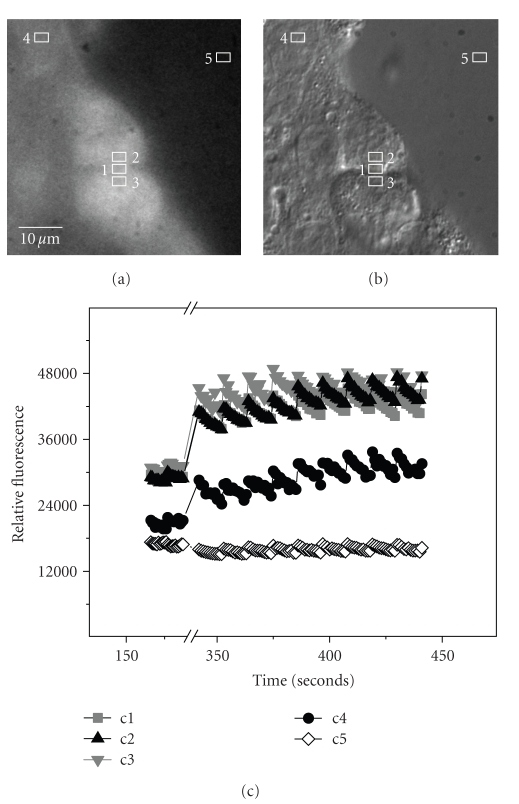
*Sites of calcium transients during mitosis*. Calcium transients correspond with sites of CaV1 immunofluorescence in AtT-20 Cells during mitosis. Simultaneous imaging of (a) Fluo4 (4 *μ*M) intracellular calcium transients and (b) cell morphology by DIC in AtT-20 cells during cytokinesis. Regions: 1=midbody; 2, 3=spindle mid-zone; 4=nuclear region of interphase cell; 5=background (media only). (c) Plot of fluorescence units for each region over time (sec). Regions of enhanced calcium transients correspond to midbody (1) and spindle mid-zone (2, 3). Break in X-axis marks time of acquisition following loading of Fluo-4.

Fura PE3/AM chelation demonstrates a clear calcium-dependence of mitosis, at least in stages prior to anaphase. However, due to this disruption, this dye does not permit calcium imaging in these cells. To image calcium transients we used Fluo-4, a calcium indicator dye with a lower affinity for free calcium. The calcium dissociation constant (K_D_) for Fura PE3/AM is 250 nM, while that for Fluo-4 is 400 nM. Using the lower affinity dye, cells are able to proceed through mitosis to allow detection of calcium fluxes at the midbody region (Figure 4(a)) as identified by simultaneous DIC imaging (Figure 4(b)), as well as in the region of the mid-spindle zone. Again, these regions correlate with the aforementioned immunocytochemical localization of CaV1.2 and CaV1.3 channels. 

 The intracellular calcium transients measured at these sites are clearly above that of the nuclear region of a nearby interphase cell (area 4) and the background (area 5) as shown in Figure 4(c). In addition, the measured elevation in intracellular calcium levels at the sites in the midbody region (areas 1–3) is unlikely to be due a “volumetric effect” of increased fluorescent probe, since the midbody and the adjacent regions are highly constricted and, therefore, represent a much smaller volume than the areas measured in the cytoplasm. Consequently, one would expect a potential under-representation, not over-representation, of calcium concentrations. Unfortunately, as shown with Fura PE3/AM above, ratiometric analysis was possible. It would be interesting to detect changes in intracellular calcium levels at more punctate sites in which CaV1s are also visualized by immunofluorescence, such as at the kinetechores during metaphase.

## 4. Discussion

Although a role for calcium in mitosis has been implicated for approximately 2 decades, the critical source of the calcium has not, to date, been demonstrated. Data in this paper provide evidence for an intriguing possible role for CaV1-type calcium channels in mitosis of AtT-20 corticotrophs. We can identify CaV1.2 immunoreactivity at the “poleward side” of the kinetechores, CaV1.3 immunofluorescence at the mid-spindle zone, and both CaV1.2 and CaV1.3 isoforms at the midbody. In addition, we are able to image calcium fluxes at sites that correspond to the mid-spindle zone (CaV1.3) and at the midbody (CaV1.2 and CaV1.3). Unfortunately, calcium imaging at other stages of mitosis is beyond the detection threshold of our current studies. However, the isoform-specific associations with discrete mitotic structures, taken together with the concomitant calcium signal intensity changes, are consistent with a functional role for these channels during cell division. Additionally, there are parallel stage-specific perturbations in the presence of a DHP antagonist as well as calcium chelation. Whereas chelation of intracellular calcium blocks mitosis at all phases prior to that of anaphase, incubation of cells with the CaV1 antagonist, nifedipine, results in a decrease in cellular proliferation that appears to be due to a “prometaphase block.” Although mechanistically different, it is interesting to note that intracellular calcium levels appear to play a pivotal role at this juncture in mitosis. 

 Evidence for a role of calcium in mitosis in at least some cell types is long-standing [13, 20]. In contrast, there is little evidence of a role for calcium channels in mitosis, although cell-cycle-dependent expression of CaV1 and CaV3 calcium currents in smooth muscle cells has been reported [37]. Stage-dependent sensitivity to changes in calcium levels has also been described. For example, Groigno and Whitaker [38] demonstrated a rise in intracellular calcium at anaphase in sea urchin embryos; this rise in calcium and the associated chromosome separation can be blocked with calcium chelation and restored with activation of caged calcium. Chandra [19] reported a redistribution of calcium stores in LLC-PK1 cells, an alignment of calcium with the mid-spindle zone prior to anaphase. 

 Given that CaVs are integral membrane proteins, our observations imply that the CaV channels must somehow transit from the cell surface to locations near mitotic structures in a phase-specific process. Many previous studies have demonstrated the importance of membrane reorganization during mitosis [37–42]. Early evidence by Pawaletz and Fehst [37] demonstrated progressive accumulation of vesicles around the mitotic apparatus in HeLa cells. During the course of mitosis vesicles are noted around chromosomes as the nuclear envelope disappears during prometaphase, over the entirety of the mitotic apparatus during metaphase, and then vesicles are detected at the mid-spindle zone and midbody, but then reaccumulate around the Golgi apparatus during telophase as it reappears during this phase. Waterman-Storer et al. [39] found membrane distribution during mitosis in both PtK2 and LLC-PK1 cells labeled with the membrane dye, DiOC6, and showed a redistribution of membrane at prophase, with tubular membrane structures aligning with the kinetechore spindles in prometaphase. Kirchhausen and colleagues have shown that mitosis is associated with a shift in the equilibrium of membrane recycling that results in a net decrease in plasma membrane and in cell size [41, 42]. This process of internalization and reorganization of plasma membrane involves SNARE-related proteins, such as Syntaxins, particularly at later stages of cell division [42–45]. Since Syntaxins and other SNARE proteins also function in exocytosis, it is provocative that our observations represent a potential parallel phenomenon, especially since there are many studies that demonstrate both direct and indirect interactions between SNARE proteins and CaV channels [6]. 

 The sites at which we observe CaV1 channels suggest a unique role for intracellular calcium prior to anaphase. For example, in metaphase there exist several “checkpoints” that regulate mitotic progression until all chromosomes are aligned at the metaphase plate and properly attached to the microtubules of the mitotic spindle. It follows, therefore, that this phase could be a sensitive stage to both nifedipine and calcium chelation, with the decrease in intracellular calcium providing a common mechanism. After metaphase, the cell proceeds to anaphase as sister chromatids are pulled apart by the mitotic spindle, a process that relies on proper microtubule attachment at the kinetochores. The presence of CaV1.3 at the mid-spindle zone is, therefore, also of interest. It is curious, however, that neither exposure to nifedipine, nor chelation of calcium, blocks the progression of anaphase in the AtT-20 cells. One possibility is that the trafficking of channels discussed above may place the targets for nifedipine in less accessible internal sites. However, the lack of effect of chelation of intracellular calcium after the initiation of anaphase also raises the possibility of parallel pathways participating at this critical juncture. 

 Finally, it is intriguing that incubation with the DHP antagonist, nifedipine, produces a decrease in cell density that is consistent with a block prior to anaphase. Previous studies of DHP effects on cellular proliferation demonstrate an inhibition of mitosis in vascular smooth muscle [29] and in sympathetic neuroblasts [26]. In PC12 cells nifedipine has also been shown to inhibit cellular differentiation [46]; this observation may be related to current visualization of CaV1.2 and CaV1.3 at the midbody in this neuroendocrine cell line. Taken together with these studies, our data provides evidence that localization of CaV channels at the mitotic apparatus may contribute to the mechanism(s) by which DHPs have been successful as adjunctive agents in chemotherapeutic regimens [47].
